# Plant Virus Differentially Alters the Plant's Defense Response to Its Closely Related Vectors

**DOI:** 10.1371/journal.pone.0083520

**Published:** 2013-12-31

**Authors:** Xiaobin Shi, Huipeng Pan, Wen Xie, Qingjun Wu, Shaoli Wang, Yang Liu, Yong Fang, Gong Chen, Xiwu Gao, Youjun Zhang

**Affiliations:** 1 Department of Plant Protection, Institute of Vegetables and Flowers, Chinese Academy of Agricultural Sciences, Beijing, P.R. China; 2 Department of Entomology, China Agricultural University, Beijing, P.R. China; Virginia Tech, United States of America

## Abstract

**Background:**

The whitefly, *Bemisia tabaci* (Hemiptera: Aleyrodidae), is one of the most widely distributed agricultural pests. In recent years, *B. tabaci* Q has invaded China, and Q has displaced B in many areas now. In a number of regions of the world, invasion by B and/or Q has been followed by outbreaks of *tomato yellow leaf curl virus* (TYLCV). Our previous study showed TYLCV directly and indirectly modified the feeding behavior of *B. tabaci* in favor of Q rather than B.

**Methodology/Principal Findings:**

In this study, we quantified the salicylic acid (SA) titers and relative gene expression of SA in tomato leaves that were infested with viruliferous or non-viruliferous B and Q. We also measured the impacts of exogenous SA on the performance of B and Q, including the effects on ovary development. SA titer was always higher in leaves that were infested with viruliferous B than with viruliferous Q, whereas the SA titer did not differ between leaves infested with non-viruliferous B and Q. The relative gene expression of SA signaling was increased by feeding of viruliferous B but was not increased by feeding of viruliferous Q. The life history traits of B and Q were adversely affected on SA-treated plants. On SA-treated plants, both B and Q had lower fecundity, shorter longevity, longer developmental time and lower survival rate than on untreated plants. Compared with whiteflies feeding on control plants, those feeding on SA-treated plants had fewer oocytes and slower ovary development. On SA-treated plants, viruliferous B had fewer oocytes than viruliferous Q.

**Conclusions/Significance:**

These results indicate that TYLCV tends to induce SA-regulated plant defense against B but SA-regulated plant defense against Q was reduced. In other words, Q may have a mutualistic relationship with TYLCV that results in the reduction of the plant's defense response.

## Introduction

Approximately 80% of plant viruses depend on insect vectors for transmission [1], [2], and the outbreak of plant viruses often depends on the abundance and distribution of their vectors. Plant-mediated interactions between pathogens and insect vectors can greatly affect the abundance of insect herbivores and the epidemiology of plant diseases [3], [4], [5]. Although much is known about the plant-virus interactions, however, less is known about plant-virus-insect interactions [6]. We are still in the early phase in understanding mechanisms of plant-mediated interactions between pathogens and herbivores, especially when the herbivores are also pathogen vectors [4], [7]. In the current study, we consider how plant responses affect the interactions between *tomato yellow leaf curl virus* (TYLCV), the vector of TYLCV (the whitefly *Bemisia tabaci*), and tomato plants.

*Bemisia tabaci* (Gennadius) (Hemiptera: Aleyrodidae) is one of the most destructive and common phloem-feeding insect pests and is a species complex composed of many biotypes that are morphologically indistinguishable [8], [9]. The two most invasive and destructive biotypes, *B. tabaci* biotype B (hereafter referred to as B) and biotype Q (hereafter referred to as Q), belong to the Middle East-Minor Asia 1 genetic group and the Mediterranean genetic group, respectively [9]. *B. tabaci* was first recorded in the late 1940s in China [10], but the crop damages and loses caused by this insect were not serious until the introduction of *B. tabaci* B in the 1990s [11]. In recent years, *B. tabaci* Q has invaded China [12], and Q has now displaced B in many areas [13], [14].

In a number of regions of the world, invasion by B and/or Q has been followed by outbreaks of TYLCV [15], [16], which is transmitted exclusively by *B. tabaci* in a circulative and persistent manner [17], [18]. TYLCV causes serious plant diseases in Africa, the Middle East, Southeast Asia, and Europe [19], [20] and, more recently, in North and South America [21], [22]. In China, TYLCV was first isolated from symptomatic tomato plants in 2006 in Shanghai [23]. Since then, it has spread to Heilongjiang, Liaoning, Neimenggu, Hebei, Beijing, Shandong, Shanxi, Jiangsu, Zhejiang, and Hubei provinces, where it has caused extensive damage to tomato crops [15]. TYLCV was not detected in China until Q became established, even though B is an important vector of TYLCV elsewhere and has been in China since the mid-1990's [14]. Previous research showed that TYLCV directly and indirectly modifies the feeding behaviors of *B. tabaci* by altering the competition between B and Q in favor of Q [24].

Plant defenses always play important roles in the interaction of insects and their vectored viruses. When feeding on virus-infected host plants, the population growth of arthropod vector species may be affected positively, negatively or neutrally [3], [25], [26]. For example, Belliure et al. (2005) [27] showed that *tomato spotted wilt virus* indirectly increase the juvenile survival and developmental rate of its thrips vector (*Frankliniella occidentalis*) through the infected host plant. However, the mechanism of how plant viruses modify the interaction of plant and its vector is still unknown. Numerous reports are often correlated with increases in salicylic acid (SA). SA mediates plant resistance to biotrophic pathogens, hemibiotrophic pathogens, and some piercing–sucking herbivores [28]. Extensive reports indicate that SA-induced defenses are important in regulating both anti-herbivore and anti-pathogen defense responses [29]–[32].

The interactions among tomato's defense responses, the whitefly, and TYLCV were examined in the current study. More specifically, we quantified endogenous SA levels and gene expression level in tomato plants infested by non-viruliferous and viruliferous B and Q and compared the performance of viruliferous and non-viruliferous B and Q on SA-treated and control tomato plants. Our goals were to determine how viruliferous and non-viruliferous vectors affect plant defense responses and how those responses affect vector performance.

## Materials and Methods

### Host plants

Tomato plants (*Lycopersicon esculentum*, cv. Zhongza 9) were grown in a potting mix (a mixture of peat moss, vermiculite, organic fertilizer, and perlite in a 10∶10∶10∶1 ratio by volume) at 25±1°C, 60±100% r.h., and L14: D10 in a glasshouse. TYLCV-infected plants were produced by *Agrobacterium tumefaciens*-mediated inoculation at the 3–4 true leaf stage with a cloned TYLCV genome (GenBank accession ID: AM282874), which was originally isolated from Shanghai, China [23]. Viral infection of test plants was confirmed by the development of characteristic leaf curl symptoms and by molecular analysis [15].

### Populations of *Bemisia tabaci* B and Q

B populations were originally collected from an infested cabbage (*Brassica oleracea*. cv. Jingfeng 1) field in Beijing, China in 2004 [15], and Q populations were originally collected from infested poinsettia (*Euphorbia pulcherrima* Wild. ex Klotz.) in Beijing, China in 2009 [15]. No specific permits were required for the described field studies. The locations for sample collection are not privately-owned or protected in any way and the field studies did not involve endangered or protected species. The B populations were maintained on cabbage and Q populations were maintained on poinsettia in screened chambers in the greenhouse. The purity of these populations was monitored by sampling 20 adults per generation using the molecular diagnostic technique CAPS (cleavage amplified polymorphic sequence) and the molecular marker mitochondrial cytochrome oxidase I gene (*mtCOI*) [13].

### Establishment of non-viruliferous and viruliferous *B. tabaci* colonies

We created four whitefly colonies: non-viruliferous B, non-viruliferous Q, viruliferous B, and viruliferous Q. We obtained viruliferous colonies by placing four TYLCV-infected tomato plants in each of two cages (60×60×60 cm). We then transferred 300 non-viruliferous B and Q adults to each of the two cages, one biotype per cage. We simultaneously established non-viruliferous B and Q colonies by placing 300 non-viruliferous B and Q adults in cages with virus-free tomato plants, one biotype per cage. All colonies were maintained for more than six generations in separate greenhouse at 25±1°C, 60±100% r.h., and L14: D10.

### Quantification of endogenous SA

Endogenous SA was quantified following the reports of Schulze et al. [33] and Matros et al. [34]. Tomato plants with 6–7 true leaves were used. Plant leaves in clip cages were treated with one of the following: non-viruliferous B, non-viruliferous Q, viruliferous B, viruliferous Q, neither B nor Q with water, or neither B nor Q with SA (as described below). Six leaves on each plant were placed in clip cages and 50 adults (or no whiteflies) were placed in the cages according to the treatments. The entire plant received the same treatment, and each treatment was represented by three parallel experiments, then the mean value was obtained as one replicate. The clip cages and the whiteflies within were removed from each plant after 0.5 h, 1 h, 1 d, 3 d, 5 d, and 7 d, and the corresponding leaves were collected at the same time; in other words, exposure time was another variable. The total experiment was repeated three times, that is to say, the free forms of SA were determined in 324 plants [6 treatments×6 incubation times×3 replicates ( = 9 plants)]. Frozen foliar tissue (0.5 g) was ground and transferred to a 5 mL microfuge tube, and 3 ml of 90% precooled methanol (90 methanol: 10 water, v/v) were added. The mixture was centrifuged at 7500 *g* for 10 min. The supernatant was transferred and the pellet was re-suspended in 2 ml of 100% methanol, then the mixture was centrifuged again at 7500 *g* for 10 min. The supernatant after twice centrifugation was then mixed and dried under vacuum, and the pellets were dissolved in 1.5 ml 5% trichloroacetic acid. After centrifugation at 7500 g for 10 min, the supernatant was extracted three times with equal volumes of ethyl acetate and cyclohexane. The organic extraction was dried, re-suspended in 3 ml of 70% methanol, loaded onto a C18 column (Waters), and then collected. After evaporation, 500 µl of acetonitrile was added and passed through a 0.45-µm filter.

All samples were analyzed by HPLC (1100; Agilent Technologies), and the fractions were collected by injecting 10 µl of the sample onto a 5-µm C18 reverse phase column (250 mm×4.6 mm; Agilent). SA was detected by excitation at 295 nm and emission at 405 nm and identified by retention time of the parallel standard SA samples. Quantitative analysis of SA was completed by plotting the results against a standard curve.

### RT-PCR gene expression analysis

Plant leaves in clip cages were treated with one of the following: non-viruliferous B, non-viruliferous Q, viruliferous B, viruliferous Q, and no whiteflies. Fifty adults (or no whiteflies) were placed in the cages according to the treatments for 1 d. Total RNA was extracted from 0.2 g of treated or control leaves, and 1 µg of RNA was used to synthesize the first-strand cDNA using the PrimeScript® RT reagent Kit (Takara Bio, Tokyo, Japan) with gDNA Eraser (Perfect Real Time, TaKara, Shiga, Japan) according to the manufacturer's protocol. To verify the genes of SA pathway were affected by infestion of *B. tabaci*, we measured the expression of the downstream genes NPR1 [35] and PR1 [36] of SA signal pathway with actin (ACT) and ubiquitin 3 (UBI) [37] as reference genes (Table 1). The 25 µl reaction system was composed of 1 µl cDNA, 12.5 µl of SYBR ® Green PCR Master Mix (TIANGEN, Corp, Beijing, China), and 0.5 ml of each primer. Relative quantities of RNA accumulation were calculated using the comparative cycle threshold (Ct) (2^−ΔΔCt^) method. Three biological replicates and four technical replicates were analysed.

**Table 1:** Primer sequences used for qPCR analysis.

Gene	Genebank Acces No.	Primer sequence
NPR1	AY 640378.1	F: 5′-ATATAGAATTCCTGCTCCAAAGGATCGGTTA-3′R: 5′-ATATACTCGAGCAGACAAGTCATCAGCATCCA-3′
PR1	AJ011520	F: 5′-ATCTCATTGTTACTCACTTGTC-3′R: 5′-AACGAGCCCGACCA-3′
ACT	BT013707	F: 5′-AGGCAGGATTTGCTGGTGATGATGCT-3′R: 5′-ATACGCATCCTTCTGTCCCATTCCGA-3′
UBI	X58253	F: 5′-TCGTAAGGAGTGCCCTAATGCTGA-3′R: 5′-CAATCGCCTCCAGCCTTGTTGTAA-3′

### Salicylic acid application

Healthy tomato plants were evenly divided into two groups: an SA-treated group and a control group. SA (Sigma-Aldrich) was dissolved in ethanol and water (1∶100, v/v) containing 10% Tween 20 to produce a 1 mM SA solution [38]. We liberally sprayed the foliage of each plant in the SA-treated group with 1.0 mL/leaf of SA solution with a hand-sprayer. The plants in the control group were sprayed with 1.0 mL/leaf of ethanol and water (1∶100, v/v) containing 10% Tween 20. The first spray was applied when the plants had six completely developed leaves, and the SA titer was determined in the first four days. According to the determination, the effect of SA was maintained by repeating the application every 3 days. Twenty-four hours after the spray, the plants were used to assess the fitness of B and Q as described in the following two sections.

### Effect of exogenous SA on life history traits of viruliferous and non-viruliferous B and Q

Nymph survivorship, developmental time, female longevity, and fecundity of non-viruliferous and viruliferous B and Q were determined on SA-treated and control tomato plants. SA-treated and control tomato plants were obtained as described in the previous section. One newly emerged female was collected and transferred to a clip-cage attached to a leaf (the third to sixth leaf from the top) of the SA-treated and control tomato plants; four clip-cages, each with one female, were attached to each plant. The eggs laid by each female were counted with a stereomicroscope (Leica, M205C) every 4 days and then the clip cages and whiteflies were transferred to new plants to maintain the effect of SA. Every female was checked daily until its death to calculate its longevity as affected by the virus status of the whiteflies and SA treatment.

Nymph survivorship (the total number of emerged adult whiteflies/the total number of eggs) and developmental time (from egg to adult) of non-viruliferous and viruliferous B and Q were measured on the SA-treated and control tomato plants. For each replicate, 10 pairs of newly emerged adults were collected and enclosed in a clip-cage with one clip-cage per tomato plant. The 10 pairs of adults in each cage were allowed to oviposit on the tomato leaf for 24 h. The adults were then removed. The leaves were then examined with a stereomicroscope (Leica, M205C), and the eggs were counted. Leaves bearing the eggs were marked. From the 16^th^ day onwards, the newly emerged adults were collected and recorded twice daily (at 9:00 and 15:00) until all the pupae had developed to adults. The total number of emerged adult whiteflies in each replicate was calculated at the end of the experiment. These data were used to calculate developmental time and survival as affected by the virus status of the whiteflies and SA treatment.

### Development of ovaries

To determine why SA greatly reduced the fecundity of B and Q (see Results), the ovaries of viruliferous whiteflies on SA-treated and control plants were compared. The experiment was carried out using the procedure as described by Guo et al. [39]. Approximately 400 pairs of newly emerged B and Q adult whiteflies were collected from TYLCV-infected tomato plants and divided into four groups with 100 pairs of adults per group. In each group, about forty adults of B or Q were used for inoculating five tomato plants which were treated by SA or water. For each treatment, 10 females were dissected every day, and the developmental phases of the oocytes in the ovaries were assessed until 15 d after eclosion.

### Statistical analysis

The survival rate of whiteflies from eggs to adults was arcsine-square root transformed for analyses. Repeated-measures ANOVAs were used to compare the quantity of endogenous SA in treated or untreated plants that were not infested with whiteflies or were infested with non-viruliferous and viruliferous B and Q. Three-way ANOVAs were used to compare the life history parameters of non-viruliferous and viruliferous B and Q on SA-treated and control tomato plants. One-way ANOVA was used to compare the oocytes of viruliferous B and Q on SA-treated and control tomato plants. One-way ANOVA was also used to compare relative gene expression of leaves infested by non-viruliferous and viruliferous B and Q compared with noninfested leaves. Means were compared by the least significant difference (LSD) test at *P*<0.05. Proportional data were arcsine square root transformed before analyses. SPSS version 20.0 (SPSS Inc., Chicago, IL, USA) was used for all statistical analyses.

## Results

### Quantification of endogenous SA

**Figure 1:**
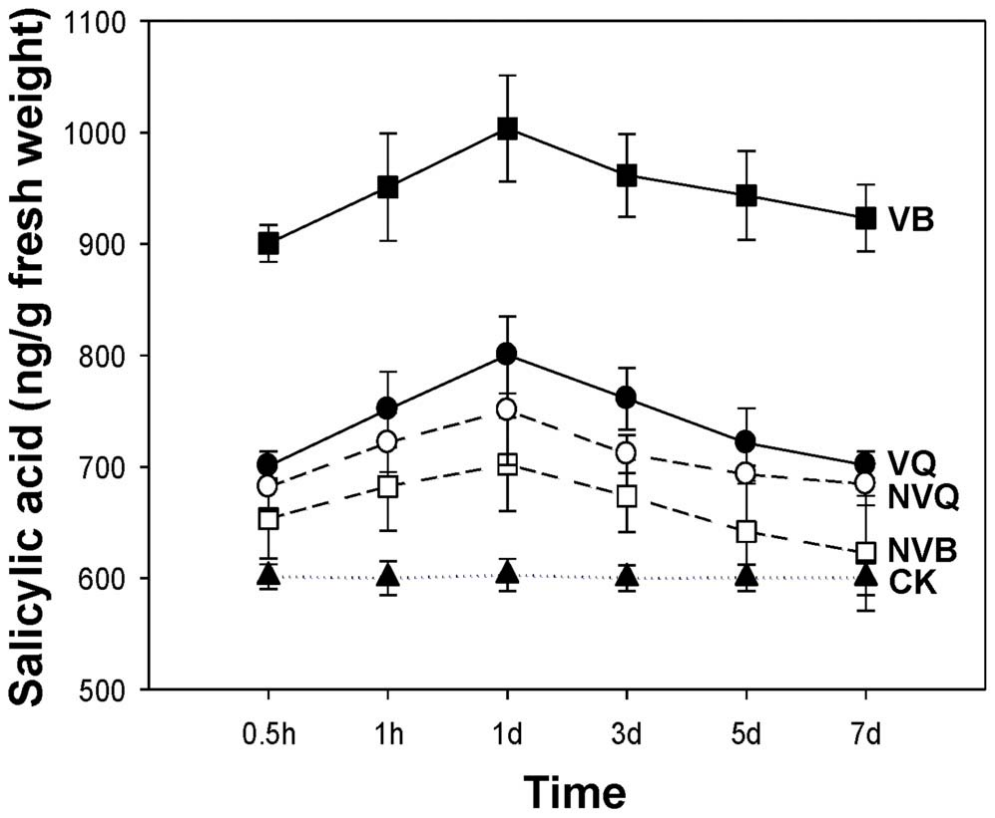
Time course of endogenous SA levels in leaves infested by non-viruliferous and viruliferous whiteflies. Non-infested leaves were used as the control. CK: control; NVQ: non-viruliferous Q; VQ: viruliferous Q; NVB: non-viruliferous B; VB: viruliferous B. Values are the means ± SE of three replicates.

**Figure 2:**
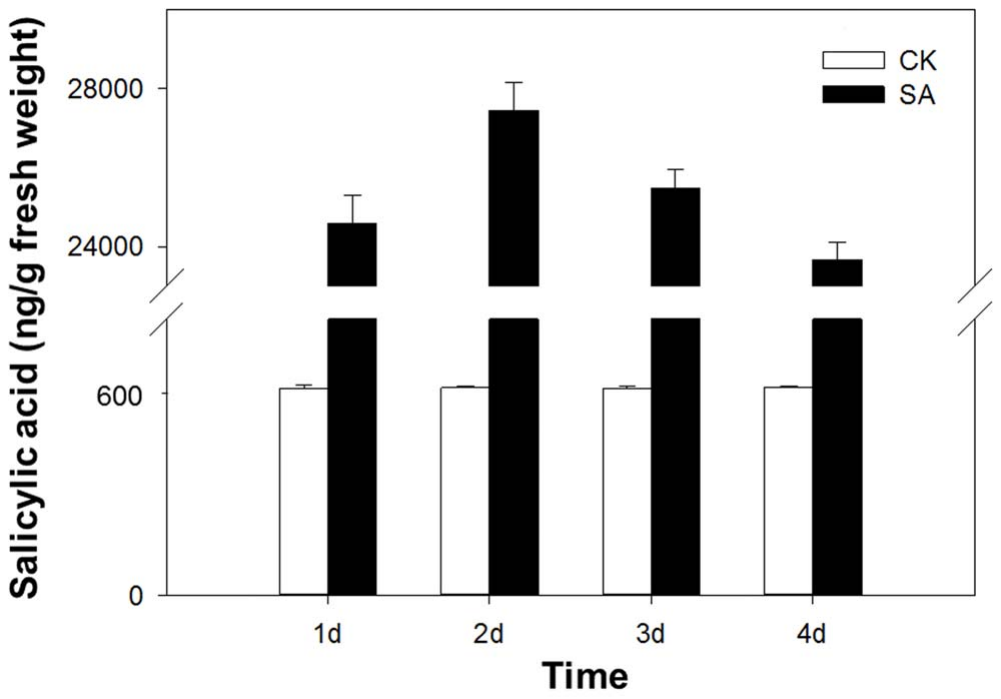
Time course of endogenous SA levels in SA-treated or untreated leaves. CK: untreated leaves; SA: SA-treated leaves. Values are the means ± SE of three replicates.

SA titers were much higher in leaves infested with viruliferous B and Q than in leaves infested with non-viruliferous B and Q (Fig. 1). SA titer in leaves was much higher with viruliferous B than with the other treatments and was lowest in the control. The differences in SA titers between leaves infested with viruliferous and non-viruliferous whiteflies was much greater for B than for Q. SA titers increased within 0.5 h after infestation, peaked after 1 d, but were still very high in the leaves infested with viruliferous B after 7 d (Fig. 1). SA titer in leaves treated with exogenous SA maintained a high level than control leaves in the first 4 days (Fig. 2), these results showed that exogenous SA-application had led to raised SA-levels, so the method of exogenous SA treatment is feasible.

### RT-PCR gene expression analysis

**Figure 3:**
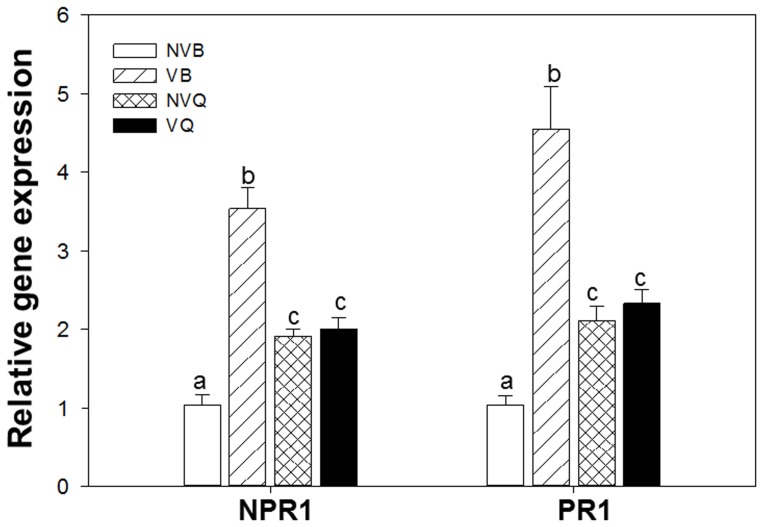
Relative gene expression in leaves infested by non-viruliferous and viruliferous whiteflies. Values were normalized to ACT and UBI. NVB: non-viruliferous B; VB: viruliferous B; NVQ: non-viruliferous Q; VQ: viruliferous Q. Values are the means ± SE of six replicates.

The relative gene expression level of NPR1 and PR1 in viruliferous B-infested leaves was significant higher than that of the nonviruliferous B-infested leaves, however, there is no significant difference between viruliferous Q-infested leaves and nonviruliferous Q-infested leaves (NPR1: *F*_3, 20_ = 35.151, *P*<0.001; PR1: *F*_3, 20_ = 23.162, *P*<0.001) (Fig. 3).

### Performance of non-viruliferous and viruliferous B and Q on SA-treated and untreated plants

**Figure 4:**
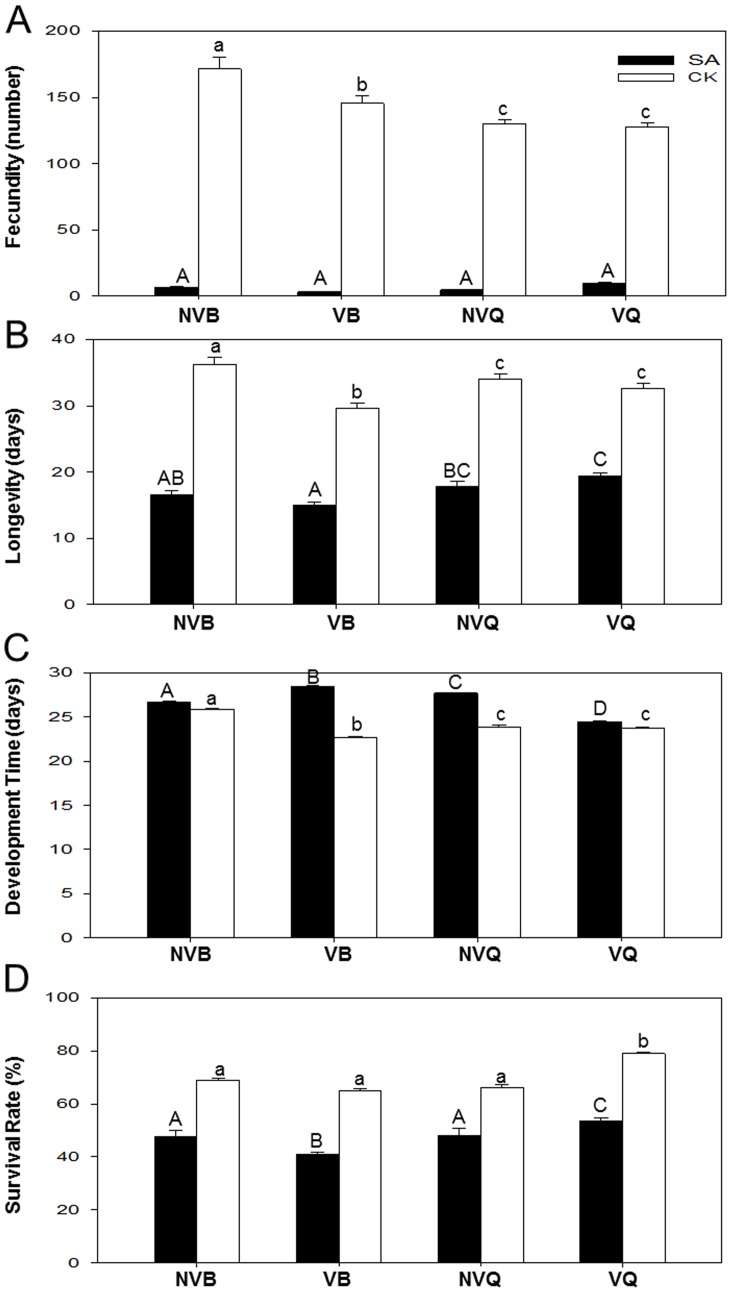
Life history traits of non-viruliferous and viruliferous whiteflies on SA-treated plants and control plants. (A) Fecundity (the total number of eggs laid by each female). (B) Longevity (from newly emerged adult until its death). (C) Developmental time (from egg to adult). (D) Survival rate (the total number of emerged adult whiteflies/the total number of eggs expressed as a percentage). NVQ: non-viruliferous Q; VQ: viruliferous Q; NVB: non-viruliferous B; VB: viruliferous B. Values are means ± SE. Different lowercase and uppercase letters indicate significant differences between treatments on control plants and SA-treated plants, respectively (LSD test at *P*<0.05).

Fecundity was significantly affected by whitefly biotype (*F*_1, 148_ = 17.530, *P*<0.001), whitefly virus status (non-viruliferous and viruliferous) (*F*_1, 148_ = 4.116, *P* = 0.044), SA treatment of plants (*F*_1, 148_ = 1789.638, *P*<0.001), and the interaction between whitefly biotype and whitefly virus status (*F*_1, 148_ = 6.302, *P* = 0.013), whitefly biotype and SA treatment of plants (*F*_1, 148_ = 24.093, *P*<0.001), and whitefly virus-status and SA treatment of plants (*F*_1, 148_ = 5.089, *P* = 0.026), but not by the interactions among the three factors (*F*_1, 148_ = 1.308, *P* = 0.255). Both B and Q laid significantly more eggs on untreated than on SA-treated plants (Fig. 4A).

Longevity was significantly affected by whitefly biotype (*F*_1, 167_ = 8.146, *P* = 0.005), whitefly virus status (*F*_1, 167_ = 12.823, *P*<0.001), SA treatment of plants (*F*_1, 167_ = 780.066, *P*<0.001), and the interaction between whitefly biotype and whitefly virus status (*F*_1, 167_ = 13.383, *P*<0.001), whitefly biotype and SA treatment of plants (*F*_1, 167_ = 4.314, *P* = 0.039), and whitefly virus-status and SA treatment of plants (*F*_1, 167_ = 11.925, *P* = 0.001), but not by the interactions among the three factors (*F*_1, 167_ = 0.624, *P* = 0.431). Longevity of both B and Q was greater on untreated than on SA-treated plants. On SA-treated plants, viruliferous Q lived longer than viruliferous B (Fig. 4B).

The mean developmental time of *B. tabaci* from egg to adult was significantly affected by whitefly biotype (*F*_1, 155_ = 140.444, *P*<0.001), whitefly virus status (*F*_1, 155_ = 217.204, *P*<0.001), SA treatment of plants (*F*_1, 155_ = 1154.592, *P*<0.001), and the interaction between whitefly biotype and whitefly virus status (*F*_1, 155_ = 33. 840, *P*<0.001), whitefly biotype and SA treatment of plants (*F*_1, 155_ = 38.526, *P*<0.001), and whitefly virus-status and SA treatment of plants (*F*_1, 155_ = 30.405, *P*<0.001), and the interactions among the three factors (*F*_1, 155_ = 564.515, *P*<0.001). Developmental time for both B and Q was shorter on untreated than on SA-treated plants. On SA-treated plants, the developmental time was longer for viruliferous B than for non-viruliferous B but was shorter for viruliferous Q than for non-viruliferous Q (Fig. 4C).

Survival rate was significantly affected by whitefly biotype (*F*_1, 103_ = 12.353, *P* = 0.001) and SA treatment (*F*_1, 103_ = 139.551, *P*<0.001) and the interaction between whitefly biotype and SA treatment (*F*_1, 103_ = 13.504, *P*<0.001) but was not affected by the whitefly virus status or interactions involving whitefly virus status. Survival for both B and Q was higher on untreated than on SA-treated plants. On SA-treated plants, survival was higher for non-viruliferous B than for viruliferous B but was lower for non-viruliferous Q than for viruliferous Q (Fig. 4D).

### Development of ovaries of viruliferous B and Q on SA-treated plants

**Figure 5:**
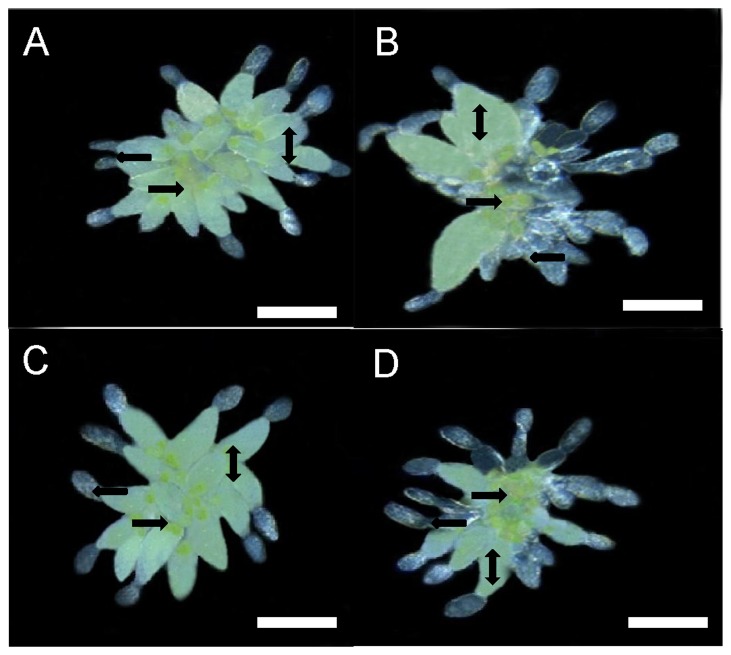
The ovaries of viruliferous whiteflies on SA-treated and control plants 14 d after eclosion. Ovaries from (A) a viruliferous B whitefly on a control plant, (B) a viruliferous B whitefly on an SA-treated plant, (C) a viruliferous Q whitefly on a control plant, and (D) a viruliferous Q whitefly on an SA-treated plant. Mature oocytes are indicated by bidirectional arrows. Immature oocytes are indicated by unidirectional leftward arrows. Bacteriocyte spheres are indicated by unidirectional rightward arrows. Scale bar: 0.10 mm.

**Figure 6:**
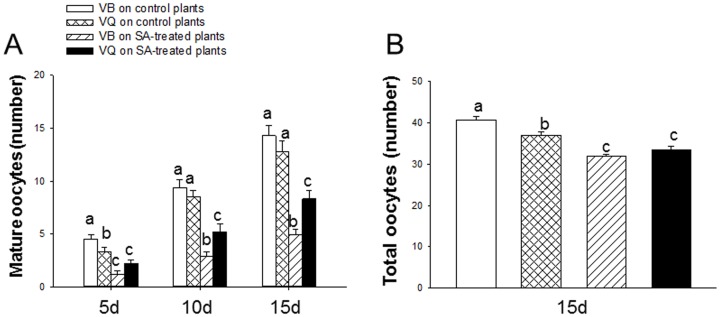
Mature and total oocytes of viruliferous whiteflies feeding on SA-treated or control plants. (A) Mature oocytes of viruliferous B and Q whiteflies on the 5^th^, 10^th^ and 15^th^ days after eclosion. Values are the means ± SE (n = 10). (B) Total oocytes of viruliferous B and Q whiteflies in the first 15 days after eclosion. Values are the means ± SE (n = 150). VB: viruliferous B; VQ: viruliferous Q. Different lowercase letters indicate significant differences (LSD test at *P*<0.05).

Whiteflies feeding on control plants (Fig. 5A and C) had a significantly more total oocytes (*F*_3, 596_ = 28.731, *P*<0.001; Fig. 6) and faster ovary development than whiteflies feeding on SA-treated plants (Fig. 5B and D; Fig. 6). On control plants, viruliferous B had significantly more total oocytes than viruliferous Q (*F*_1, 298_ = 0.268, *P* = 0.001) (Fig. 5A and C; Fig. 6). On SA-treated plants, viruliferous B had fewer oocytes than viruliferous Q (*F*_1, 298_ = 15.102, *P* = 0.089) (Fig. 5B and D; Fig. 6).

## Discussion

Insect herbivores and microbial pathogens may manipulate plant defense responses for their own benefits [40], [41]. For insect vectors, the effect of virus can be direct or indirect. Gutiérrez et al. (2013) reported that plant viruses can influence vector physiology and behavior so as to increase virus transmission either directly or through modification of the host plant [6]. For example, Moreno-Delafuente et al (2013) found that TYLCV can directly modify the behavior of its insect vector, *B. tabaci* to facilitate its own transmission [42]. In some cases, virus transmission by insect vectors can be increased through virus-induced changes in the plant [43], and the indirect interaction reflects a plant-mediated mutualistic relationship between vectors and pathogens [3], [4]. In the present study, we demonstrated that the fecundity, longevity and developmental time was similar for viruliferous Q and non-viruliferous Q but was lower for viruliferous B than for non-viruliferous B. This result is consistent with our previous finding that Q is a better vector of TYLCV than B and that the spread of TYLCV in China is closely related with the rapid establishment and spread of Q [15]. Our results showed that the growth and development of B and Q was differently affected by virus through different defense response of plants.

A number of studies have shown that phloem-feeding insects, such as aphids and whiteflies, induce SA-dependent responses [44]–[47]. SA can have neutral or negative effects on the growth of aphids and whiteflies [48]. Transcriptome analyses indicated that feeding by these insects elicits SA-regulated transcripts [49]–[51]. Avila et al. (2012) [35] showed that FAD7 enhances plant defenses against aphids that are mediated through SA and NPR1. In our research we also found that the relative expression of NPR1 and PR1 were induced by the feeding of whiteflies. There is a great similarity between pathogen- and herbivore-induced signal events. For example, Huang et al. (2012) [52] reported that TYLCV infection significantly increased SA levels in tomato plants. Abe et al. (2011) [53] demonstrated that *tomato spotted wilt virus* infection elevated SA contents and induced SA-regulated gene expression in *Arabidopsis* plants. Rodriguez-Saona et al. (2010) [54] showed that the SA-mediated defense responses are effective against both pathogens and aphids in tomato, because *tobacco mosaic virus* infection reduces plant susceptibility to aphids in wild-type tomato but not in SA-deficient transgenic plants.

In our current study, the SA titer in leaves was higher when they were infested with viruliferous B or Q than with non-viruliferous B or Q, although the difference was relatively small for Q but large for B. The similar trend was determined on the relative gene expression in SA signaling pathway. These results indicate that TYLCV and *B. tabaci* infection simultaneously increased the endogenous SA levels and induced the SA-regulated defense system. In our research the basal SA-levels in control plants are very high in comparison to other published studies [35], [53], the possible reason is that there are differences between plants, besides, different light, temperature, humidity and experiment conditions may also be associated with the SA titer.

To combat pathogens and insect herbivores, plants have evolved sophisticated mechanisms that ensure early detection and induction of appropriate defense responses [55]. At the same time, pathogens and herbivores have evolved mechanisms to evade or suppress host resistance [56], [57]. The plant evidently uses the SA signal to inform plant components that attack is imminent or ongoing. In the current study, the SA titer was much higher in leaves infested with viruliferous B than with viruliferous Q, while the SA titer was not very different in leaves infested with non-viruliferous B and Q. From such results we can conclude that the defense response of plants induced by viruliferous B maybe stronger than that induced by viruliferous Q. In other words, viruliferous Q may be better able than viruliferous B to reduce the plant's defense response. More experiments are required to reveal why host plants respond differently to these and other viruliferous vectors.

Vectors often perform better on plants infected with pathogens, and this promotes the spread of pathogens. One possible mechanism is that many herbivores have acquired traits, often in the form of secreted substances, those interfere with the plant's ability to organize its defenses. For example, the fungal pathogen *Fusarium oxysporum* releases “effectors” that specifically interfere with the plant's defense hormone signaling [58], and the bacterial pathogen *Pseudomonas syringae* DC3000 [59] uses the plant's JA-SA defenses to its own advantage. Similarly, the saliva of some aphids [60] contains proteins that prevent feeding site occlusion [61]. Perhaps the difference of whitefly-secreted substances explains the different defense responses to viruliferous B vs. viruliferous Q. Another possibility to consider is that fatty acids have been proposed to participate in defense signaling either directly or indirectly [62] and are also precursors for the synthesis of azelaic acid and numerous oxylipins that contribute to plant immunity [63]. The endosymbiotic bacteria may also play an important role in the interaction. As we know, the transmission of viruses by *B. tabaci* to plants is related to a protein created by an endosymbiotic bacteria [64]. Previous research showed that at the same time, the virus titers harbored in the body of Q is higher than that in B [15]. As we know, virus can also induce the SA defense, but the SA titer and expression of VQ is lower than that of VB (Fig. 1 and Fig. 3). We speculate the different distribution of endosymbiotic bacterias between B and Q contributes to this difference [65].

Plant defenses often affect whitefly activity and performance [40]. In the present study, both B and Q had reduced fecundity, reduced longevity, slower development, and lower survival rates on SA-treated plants than on non-treated plants. In addition, viruliferous B performed worse than viruliferous Q on SA-treated tomato plants. Previous research showed that SA was an effective chemical defense response against aphids [66]. However, Zarate et al. (2007) [40] showed that whitefly nymphs were able to feeding and growth well on up-regulation of SA-dependent defense. One possible reason is that there may be some difference between nymphs and adults because nymphs have a long-term interaction with their host plants. Another possible reason is that different amount of SA may have different effect. The SA treatment in our research may trigger much stronger defense which is different from the physiological defense, therefore the exogenous defense activated by spaying of SA maybe different from endogenous SA-dependent defense.

In conclusion, tomato plants responded to *B. tabaci* attack by activating the endogenous SA-regulated defenses, and the response was stronger against B than Q. Application of SA decreased the performance of both B and Q but this effect was modified by TYLCV, i.e., the negative effect of SA tended to be enhanced for B but reduced for Q. The results suggest that *B. tabaci* Q has a mutualistic relationship with TYLCV that results in the suppression of the plant's defense response. The possible reason is that the secreted substances or endosymbiotic bacterias of *B. tabaci* were different changed by virus, and this may help to change SA pathway of plant defense. Additional research is required to clarify the nature of this three-way interaction and of other plant–virus–vector interactions. Such research should enhance the development of crop protection strategies.

## References

[1] Hohn T (2007) Plant virus transmission from the insect point of view. Proc Natl Acad Sci U S A 104: 17905–17906. 17989216 10.1073/pnas.0709178104 PMC2084268

[2] Andret-Link P, Fuchs M (2005) Transmission specificity of plant viruses by vectors. J Plant Pathol 87: 153–165.

[3] Colvin J, Omongo CA, Govindappa MR, Stevenson PC, Maruthi MN, et al (2006) Host-plant viral infection effects on arthropod-vector population growth, development and behaviour: management and epidemiological implications. Adv Virus Res 67: 419–452. 17027686 10.1016/S0065-3527(06)67011-5

[4] Stout MJ, Thaler JS, Thomma BPHJ (2006) Plant-mediated interactions between pathogenic microorganisms and herbivorous arthropods. Annu Rev Entomol 51: 663–689. 16332227 10.1146/annurev.ento.51.110104.151117

[5] Mauck K, Bosque-Pérez NA, Eigenbrode SD, De Moraes CM, Mescher MC (2012) Transmission mechanisms shape pathogen effects on host–vector interactions: evidence from plant viruses. Funct Ecol 26: 1162–1175.

[6] Gutiérrez S, Michalakis Y, van Munster M, Blanc S (2013) Plant feeding by insect vectors can affect life cycle, population genetics and evolution of plant viruses. Funct Ecol 27: 610–622.

[7] Pieterse CMJ, Dicke M (2007) Plant interactions with microbes and insects: from molecular mechanisms to ecology. Trends Plant Sci 12: 564–568. 17997347 10.1016/j.tplants.2007.09.004

[8] Brown JK, Frohlich DR, Rosell RC (1995) The sweetpotato or silverleaf whiteflies: biotypes of *Bemisia tabaci* or a species complex? Annu Rev Entomol 40: 511–534.

[9] De Barro PJ, Liu SS, Boykin LM, Dinsdale AB (2011) *Bemisia tabaci*: a statement of species status. Annu Rev Entomol 56: 1–19. 20690829 10.1146/annurev-ento-112408-085504

[10] Zhou Y (1949) The list of whiteflies in China. China Entomol 3: 1–18.

[11] Luo C, Yao Y, Wang RJ, Yan FM, Hu DX, et al (2002) The use of mitochondrial cytochrome oxidase mtCOI gene sequences for the identification of biotypes of *Bemisia tabaci* (Gennadius) in China. Acta Entomol Sin 45: 759–763.

[12] Chu D, Zhang YJ, Brown JK, Cong B, Xu BY, et al (2006) The introduction of the exotic Q biotype of *Bemisia tabaci* from the mediterranean region into China on ornamental crops. Fla Entomol 89: 168–174.

[13] Chu D, Wan FH, Zhang YJ, Brown JK (2010) Change in the biotype composition of *Bemisia tabaci* in shandong province of China from 2005 to 2008. Environ Entomol 39: 1028–1036. 20550819 10.1603/EN09161

[14] Pan HP, Chu D, Ge DQ, Wang SL, Wu QJ, et al (2011) Further spread of and domination by *Bemisia tabaci* (Hemiptera: Aleyrodidae) biotype Q on field crops in China. J Econ Entomol 104: 978–985. 21735919 10.1603/ec11009

[15] Pan HP, Chu D, Yan WQ, Su Q, Liu BM, et al (2012) Rapid spread of *tomato yellow leaf curl virus* in China is aided differentially by two invasive whiteflies. PLoS One 7: e34817. 22514670 10.1371/journal.pone.0034817 PMC3325912

[16] Navas-Castillo J, Fiallo-Olivé E, Sánchez-Campos S (2011) Emerging virus diseases transmitted by whiteflies. Annu Rev Phytopathol 49: 219–248. 21568700 10.1146/annurev-phyto-072910-095235

[17] Cohen S, Nitzany FE (1966) Transmission and host range of the *tomato yellow leaf curl virus* . Phytopathology 56: 1127–1131.

[18] Rubinstein G, Czosnek H (1997) Long-term association of *tomato yellow leaf curl virus* with its whitefly vector *Bemisia tabaci*: effect on the insect transmission capacity, longevity and fecundity. J Gen Virol 78: 2683–2689. 9349491 10.1099/0022-1317-78-10-2683

[19] Czosnek H, Laterrot H (1997) A worldwide survey of *tomato yellow leaf curl viruses* . Arch Virol 142: 1391–1406. 9267451 10.1007/s007050050168

[20] Moriones E, Navas-Castillo J (2000) *Tomato yellow leaf curl virus*, an emerging virus complex causing epidemics worldwide. Virus Res 71: 123–134. 11137167 10.1016/s0168-1702(00)00193-3

[21] Polston JE, McGovern RJ, Brown LG (1999) Introduction of *tomato yellow leaf curl virus* in Florida and implications for the spread of this and other geminiviruses of tomato. Plant Dis 83: 984–988. 10.1094/PDIS.1999.83.11.984 30841296

[22] Zambrano K, Carballo O, Geraud F, Chirinos D, Fernández C, et al (2007) First report of *tomato yellow leaf curl virus* in Venezuela. Plant Dis 91: 768. 10.1094/PDIS-91-6-0768A 30780492

[23] Wu JB, Dai FM, Zhou XP (2006) First report of *tomato yellow leaf curl virus* in China. Ann Appl Biol 155: 439–448. 10.1094/PD-90-1359C 30780951

[24] Liu BM, Preisser EL, Chu D, Pan HP, Xie W, et al (2013) Multiple forms of vector manipulation by a plant-infecting virus: *Bemisia tabaci* and *tomato yellow leaf curl virus* . J Virol 87 9: 4929. 23408638 10.1128/JVI.03571-12 PMC3624301

[25] Whitfield AE, Ullman DE, German TL (2005) Tospovirus-thrips interactions. Annu Rev Phytopathol 43: 459–489. 16078892 10.1146/annurev.phyto.43.040204.140017

[26] Goggin FL (2007) Plant–aphid interactions: molecular and ecological perspectives. Curr Opin Plant Biol 10: 399–408. 17652010 10.1016/j.pbi.2007.06.004

[27] Belliure B, Janssen A, Maris PC, Peters D, Sabelis MW (2005) Herbivore arthropods benefit from vectoring plant viruses. Ecol Lett 8: 70–79.

[28] Walling LL (2009) Adaptive defense responses to pathogens and insects. Adv Bot Res 51: 551–612.

[29] Delaney TP, Uknes S, Vernooij B, Friedrich L, Weymann K, et al (1994) A central role of salicylic acid in plant disease resistance. Science 266: 1247–1250. 17810266 10.1126/science.266.5188.1247

[30] Glazebrook J (2005) Contrasting mechanisms of defense against biotrophic and necrotrophic pathogens. Annu Rev Phytopathol 43: 205–227. 16078883 10.1146/annurev.phyto.43.040204.135923

[31] Erb M, Meldau S, Howe GA (2012) Role of phytohormones in insect-specific plant reactions. Trends Plant Sci 17: 250–259. 22305233 10.1016/j.tplants.2012.01.003 PMC3346861

[32] Yang JW, Yi HS, Kim H, Lee B, Lee S, et al (2011) Whitefly infestation of pepper plants elicits defence responses against bacterial pathogens in leaves and roots and changes the below-ground microflora. J Ecol 99: 46–56.

[33] Schulze B, Lauchli R, Sonwa MM, Schmidt A, Boland W (2006) Profiling of structurally labile oxylipins in plants by in situ derivatization with pentafluorobenzyl hydroxyl-amine. Anal Biochem 348: 269–283. 16307716 10.1016/j.ab.2005.10.021

[34] Matros A, Amme S, Kettig B, Buck-Sorlin GH, Sonnewald U, et al (2006) Growth at elevated CO_2_ concentrations leads to modified profiles of secondary metabolites in tobacco cv. SamsunNN and to increased resistance against infection with Potato virus Y. Plant Cell Environ 29: 126–137. 17086759 10.1111/j.1365-3040.2005.01406.x

[35] Avila CA, Arevalo-Soliz LM, Jia LL, Navarre DA, Chen Z, et al (2012) Loss of function of *FATTY ACID DESATURASE7* in tomato enhances basal aphid resistance in a salicylate-dependent manner. Plant Physiol 158: 2028–2041. 22291202 10.1104/pp.111.191262 PMC3320204

[36] Peng JY, Deng XJ, Huang JH, Jia SH, Miao XX, et al (2004) Role of salicylic acid in tomato defense against cotton bollworm, *Helicoverpa armigera* Hubner. Z Naturforsch C 59: 856–862. 15666546 10.1515/znc-2004-11-1215

[37] Mascia T, Santovito E, Gallitelli D, Cillo F (2010) Evaluation of reference genes for quantitative reverse-transcription polymerase chain reaction normalization in infected tomato plants. Mol Plant Pathol 11: 805–816. 21029324 10.1111/j.1364-3703.2010.00646.x PMC6640390

[38] Wu W, Ding Y, Wei W, Davis RE, Lee IM, et al (2012) Salicylic acid-mediated elicitation of tomato defence against infection by potato purple top phytoplasma. Ann Appl Biol 161: 36–45.

[39] Guo JY, Ye GY, Dong SZ, Liu SS (2010) An invasive whitefly feeding on a virus-infected plant increased its egg production and realized fecundity. PLoS One 5: e11713. 20676356 10.1371/journal.pone.0011713 PMC2911204

[40] Zarate SI, Kempema LA, Walling LL (2007) Silverleaf whitefly induces salicylic acid defenses and suppresses effectual jasmonic acid defenses. Plant Physiol 143: 866–875. 17189328 10.1104/pp.106.090035 PMC1803729

[41] Sarmento RA, Lemos F, Bleeker PM, Schuurink RC, Pallini A, et al (2011) A herbivore that manipulates plant defence. Ecol Lett 14: 229–236. 21299823 10.1111/j.1461-0248.2010.01575.x PMC3084520

[42] Moreno-Delafuente A, Garzo E, Moreno A, Fereres A (2013) A plant virus manipulates the behavior of its whitefly vector to enhance its transmission efficiency and spread. PLoS ONE 8: e61543. 23613872 10.1371/journal.pone.0061543 PMC3629040

[43] Bosque-Perez NA, Eigenbrode SD (2011) The influence of virus-induced changes in plants on aphid vectors: insights from luteovirus pathosystems. Virus Res 159: 201–205. 21549769 10.1016/j.virusres.2011.04.020

[44] De Vos M, Van Oosten VR, Van Poecke RM, Van Pelt JA, Pozo MJ, et al (2005) Signal signature and transcriptome changes of *Arabidopsis* during pathogen and insect attack. Mol Plant Microbe Interact 18: 923–937. 16167763 10.1094/MPMI-18-0923

[45] Walling LL (2000) The myriad plant responses to herbivores. J Plant Growth Regul 19: 195–216. 11038228 10.1007/s003440000026

[46] Thaler JS, Agrawal AA, Halitschke R (2010) Salicylate-mediated interactions between pathogens and herbivores. Ecology 91: 1075–1082. 20462121 10.1890/08-2347.1

[47] Blande JD, Korjus M, Holopainen JK (2010) Foliar methyl salicylate emissions indicate prolonged aphid infestation on silver birch and black alder. Tree Physiol 30: 404–416. 20097686 10.1093/treephys/tpp124

[48] Pegadaraju V, Knepper C, Reese J, Shah J (2005) Premature leaf senescence modulated by the *Arabidopsis PHYTOALEXIN DEFICIENT4* gene is associated with defense against the phloem-feeding green peach aphid. Plant Physiol 139: 1927–1934. 16299172 10.1104/pp.105.070433 PMC1310570

[49] Moran PJ, Thompson GA (2001) Molecular responses to aphid feeding in *Arabidopsis* in relation to plant defense pathways. Plant Physiol 125: 1074–1085. 11161062 10.1104/pp.125.2.1074 PMC64906

[50] Moran PJ, Cheng Y, Cassell JL, Thompson GA (2002) Gene expression profiling of *Arabidopsis thaliana* in compatible plant–aphid interactions. Arch Insect Biochem Physiol 51: 182–203. 12432519 10.1002/arch.10064

[51] De Vos M, Kim JH, Jander G (2007) Biochemistry and molecular biology of *Arabidopsis*–aphid interactions. BioEssays 29: 871–883. 17691101 10.1002/bies.20624

[52] Huang LC, Ren Q, Sun YC, Ye LF, Cao HF, et al (2012) Lower incidence and severity of tomato virus in elevated CO_2_ is accompanied by modulated plant induced defense in tomato. Plant Biology 14: 905–913. 22512888 10.1111/j.1438-8677.2012.00582.x

[53] Abe H, Tomitaka Y, Shimoda T, Seo S, Sakurai T, et al (2012) Antagonistic plant defense system regulated by phytohormones assists interactions among vector insect, thrips and a tospovirus. Plant Cell Physiol 53: 204–212. 22180600 10.1093/pcp/pcr173

[54] Rodriguez-Saona CR, Musser RO, Vogel H, Hum-Musser SM, Thaler JS (2010) Molecular, biochemical, and organism alanalyses of tomato plants simultaneously attacked by herbivores from two feeding guilds. J Chem Ecol 36: 1043–1057. 20820890 10.1007/s10886-010-9854-7

[55] De Vos M, Jander G (2009) *Myzus persicae* (green peach aphid) salivary components induce defence responses in *Arabidopsis thaliana* . Plant Cell Environ 32: 1548–1560. 19558622 10.1111/j.1365-3040.2009.02019.x

[56] Barrett LG, Heil M (2012) Unifying concepts and mechanisms in the specificity of plant–enemy interactions. Trends Plant Sci 17: 282–292. 22465042 10.1016/j.tplants.2012.02.009

[57] Alba JM, Glas JJ, Schimmel BCJ, Kant M (2011) Avoidance and suppression of plant defenses by herbivores and pathogens. J Plant Interact 6: 1–7.

[58] Thatcher LF, Manners JM, Kazan K (2009) *Fusarium oxysporum* hijacks COI1-mediated jasmonate signaling to promote disease development in *Arabidopsis* . Plant J 58: 927–939. 19220788 10.1111/j.1365-313X.2009.03831.x

[59] Katsir L, Schilmiller AL, Staswick PE, He SY, Howe GA (2008) COI1 is a critical component of a receptor for jasmonate and the bacterial virulence factor coronatine. Proc Natl Acad Sci U S A 105: 7100–7105. 18458331 10.1073/pnas.0802332105 PMC2383947

[60] Carolan JC, Fitzroy CIJ, Ashton PD, Douglas AE, Wilkinson TL (2009) The secreted salivary proteome of the pea aphid *Acyrthosiphon pisum* characterised by mass spectrometry. Proteomics 9: 2457–2467. 19402045 10.1002/pmic.200800692

[61] Giordanengo P, Brunissen L, Rusterucci C, Vincent C, van Bel A, et al (2010) Compatible plant-aphid interactions: how aphids manipulate plant responses. CR Biol 333: 516–523. 10.1016/j.crvi.2010.03.007 20541163

[62] Kachroo A, Kachroo P (2009) Fatty acid-derived signals in plant defense. Annu Rev Phytopathol 47: 153–176. 19400642 10.1146/annurev-phyto-080508-081820

[63] Jung HW, Tschaplinski TJ, Wang L, Glazebrook J, Greenberg JT (2009) Priming in systemic plant immunity. Science 324: 89–91. 19342588 10.1126/science.1170025

[64] Gottlieb Y, Zchori-Fein E, Mozes-Daube N, Kontsedalov S, Skaljac M, et al (2010) The transmission efficiency of *tomato yellow leaf curl virus* by the whitefly *Bemisia tabaci* is correlated with the presence of a specific symbiotic bacterium species. J Virol 84: 9310–9317. 20631135 10.1128/JVI.00423-10 PMC2937599

[65] Pan HP, Li XC, Ge DQ, Wang SL, Wu QJ, et al (2012) Factors affecting population dynamics of maternally transmitted endosymbionts in *Bemisia tabaci* . PLoS ONE 7: e30760. 22383972 10.1371/journal.pone.0030760 PMC3285672

[66] Donovan MP, Nabity PD, DeLucia EH (2013) Salicylic acid-mediated reductions in yield in *Nicotiana attenuate* challenged by aphid herbivory. Arthropod-Plant Inte 7: 45–52.

